# Modeling the current and future habitat suitability of *Neltuma pallida* in the dry forest of northern Peru under climate change scenarios to 2100

**DOI:** 10.1002/ece3.70158

**Published:** 2024-08-27

**Authors:** Elgar Barboza, Nino Bravo, Alexander Cotrina‐Sanchez, Wilian Salazar, David Gálvez‐Paucar, Jhony Gonzales, David Saravia, Lamberto Valqui‐Valqui, Gloria P. Cárdenas, Jimmy Ocaña, Juancarlos Cruz‐Luis, Carlos I. Arbizu

**Affiliations:** ^1^ Dirección de Supervisión y Monitoreo en las Estaciones Experimentales Agrarias Instituto Nacional de Innovación Agraria (INIA) Lima Peru; ^2^ Laboratorio de Agrostología Instituto de Investigación en Ganadería y Biotecnología Universidad Nacional Toribio Rodríguez de Mendoza de Amazonas (UNTRM) Chachapoyas Peru; ^3^ Estación Experimental Agraria Pucallpa Instituto Nacional de Innovación Agraria (INIA) Pucallpa Peru; ^4^ Instituto de Investigación para el Desarrollo Sustentable de Ceja de Selva Universidad Nacional Toribio Rodríguez de Mendoza de Amazonas (UNTRM) Chachapoyas Peru; ^5^ Department for Innovation in Biological, Agri‐Food and Forest Systems Università Degli Studi Della Tuscia Viterbo Italy; ^6^ Dirección de Desarrollo Tecnológico Agrario Instituto Nacional de Innovación Agraria (INIA) Lima Peru; ^7^ Instituto de Investigación en Desarrollo Sostenible y Cambio Climático Universidad Nacional de Frontera (UNF) Sullana Peru; ^8^ Present address: Facultad de Ingeniería y Ciencias Agrarias Universidad Nacional Toribio Rodríguez de Mendoza de Amazonas (UNTRM) Chachapoyas Peru

**Keywords:** biodiversity, coastal dry forest, google earth engine, habitat, MaxEnt

## Abstract

The development of anthropic activities and climate change effects impact worldwide species' ecosystems and habitats. Habitats' adequate prediction can be an important tool to assess current and future trends. In addition, it allows strategies development for their conservation. The *Neltuma pallida of* the forest region in northern Peru, although very significant, has experienced a decline in recent years. The objective of this research is to evaluate the current and future distribution and conservation status of *N. pallida* in the Peruvian dry forest under climate change (Location: Republic of Peru). A total of 132 forest presence records and 10 variables (bioclimatic, topographic, and soil) were processed and selected to obtain the current and future distribution for 2100, using Google Earth Engine (GEE), RStudio, and MaxEnt. The area under the curve values fell within the range of 0.93–0.95, demonstrating a strong predictive capability for both present and future potential habitats. The findings indicated that the likely range of habitats for *N. pallida* was shaped by factors such as the average temperature of wettest quarter, maximum temperature of warmest month, elevation, rainfall, and precipitation of driest month. The main suitable areas were in the central regions of the geographical departments of Tumbes, Piura, and Lambayeque, as well as in the northern part of La Libertad. It is critical to determine the habitat suitability of plant species for conservation managers since this information stimulates the development of policies that favor sustainable use programs. In addition, these results can contribute significantly to identify new areas for designing strategies for populations conserving and recovering with an ecological restoration approach.

## Graphical abstract

The objective of this research was to evaluate the current and future distribution and conservation status of *Neltuma pallida* in the Peruvian dry forest under climate change scenarios over the last few years. The findings indicated that the likely range of habitats for *N. pallida* was shaped by factors such as the average temperature of wettest quarter, maximum temperature of warmest month, elevation, rainfall, and precipitation of driest month. The main suitable areas were located in the central regions of the departments of Tumbes, Piura, and Lambayeque, as well as in the northern part of the department of La Libertad.

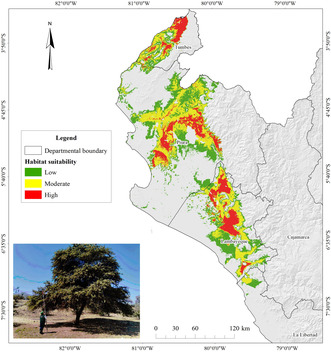

## 1 INTRODUCTION

*Neltuma pallida* is a tree, 8–20 m in height, belonging to the *Neltuma genus* (Subfamily: Caesalpinioideae). It is distributed in arid and dry tropical regions of the Americas (Hughes et al., 2022). This species is native to arid areas of Peru, Ecuador, and Colombia (Burkart, 1981). In Peru, this species is found in the Equatorial Dry Forest (3.45% of the country's surface area), representing 61% of the entire vegetation cover of the dry (Salazar, Navarro‐Cerrillo, Ancajima, et al., 2018; Salazar, Navarro‐Cerrillo, Cruz, & Villar, 2018), between the departments of La Libertad, Lambayeque, Piura, and Tumbes. It holds economic value as it is used as firewood and charcoal for fuel in rural communities (Caycho et al., 2023; OSINFOR, 2018). Additionally, *N. pallida* has ecological value by providing ecosystem services such as controlling water erosion, soil fertility, climate regulation, and bioremediation (Ambite et al., 2022; Caycho et al., 2023; Mokgalaka‐Matlala et al., 2009; OSINFOR, 2018; Santos‐Jallath et al., 2012; SERFOR, 2021). This species is severely threatened, and the causes so far are uncertain and likely complex (Caycho et al., 2023). In the dry forest of Peru, *N. pallida* populations are experiencing a decline of up to 49% (SERFOR, 2020); climate change, drought, pests, and diseases have influenced its decline (Salazar, Navarro‐Cerrillo, Ancajima, et al., 2018; Salazar, Navarro‐Cerrillo, Cruz, & Villar, 2018; SERFOR, 2021).

Climate change can have a significant impact by altering the composition of terrestrial ecosystem communities and the performance of species (Bertrand et al., 2011; Forrest, 2016). It often affects the geographic distribution area of endangered species and reduces the size of their native habitats, ultimately resulting in a decline in population or even extinction (Anderegg et al., 2019; Khanal et al., 2022). However, this will depend on the habitat of each species (Bertin, 2008; Meir et al., 2015). The El Niño‐Southern Oscillation (ENSO) alters global precipitation patterns, increasing temperatures in arid areas with less frequent rainfall under normal conditions, and temperatures may be more moderate. El Niño‐Southern Oscillation indices, Pacific indices, Walker circulation, and the Humboldt Current are factors that control air temperature and climate on the northern coast of Peru (Rollenbeck et al., 2015). ENSO on the northern coast of Peru has benefited desert vegetation by improving primary productivity levels (Vining et al., 2022) and reducing rural community poverty by 5% in this ecosystem (Pécastaing & Chávez, 2020).

Species distribution modeling (SDM) is an important tool for research because they provide spatial information on the current and future environmental suitability of species (Elith & Leathwick, 2009; Mammola et al., 2021; Santini et al., 2021; Wang et al., 2020). Species distribution modeling defines the species–environment relationship to estimate the geographical distribution of species under different climatic scenarios (Gobeyn et al., 2019). A variety of SDM has been developed (Climex, Genetic Algorithm for Rule‐Set Production [GARP], BIOCLIM, and MaxEnt); however, comparative studies suggest that the MaxEnt model is the most suitable (Elith et al., 2011; Liu et al., 2021; Phillips & Dudík, 2008). In dry forest ecosystems, the potential distributions of current and future habitats of species such as *Cavanillesia platanifolia*, *Cordia*, *Erythrina velutina*, *Handroanthus chrysanthus*, *Terminalia valverdeae*, *Euterpe edulis Mart*, *Prosopis juliflora*, and *Prosopis pallida* (now *N. juliflora* and *N. pallida*) have been studied. Some of these habitats decrease under climate change, while others increase (Aguirre et al., 2017; Leal et al., 2022; Oliveira et al., 2018).

*Neltuma pallida* forms extensive forests in northern Peru (Zorogastúa et al., 2011). It provides multiple ecosystem benefits as an environment preserver, soil protector and fertilizer, and as a food source for goat farming (Cruzado‐Jacinto et al., 2019). In addition, it is used in construction and firewood for populations settled in dry forest ecosystems (Cuentas & Salazar, 2017). However, this species has been reduced by deforestation, urbanization, and agricultural expansión (Depenthal & Meitzner, 2017).

Studies of *N. pallida* have focused mainly on evaluating its physiological characteristics and its ecological and economic valuation (Aguirre & Kvist, 2005; Cuentas Romero, 2015; Espinosa et al., 2012). Others are related to the insect's identification associated with the species and its biodiversity (Cruzado‐Jacinto et al., 2019; SERFOR et al., 2022), other researchers evaluated foliar functional traits and adaptability to extreme climatic events (Salazar et al., 2021; Salazar, Navarro‐Cerrillo, Ancajima, et al., 2018; Salazar, Navarro‐Cerrillo, Cruz, & Villar, 2018). There have been reports of studies with limited data regarding the present and future ecological suitability distribution of *N. pallida*. Hence, studying the behavior of its habitat with respect to the effect of climate change under different scenarios is necessary.

Information on the distribution of *N. pallida*, spatial arrangement, and climatically suitable habitats under current and future conditions can be assessed by using tools such as QGIS, RStudio, and MaxEnt (Bushi et al., 2022; Kalboussi & Achour, 2018; Shi et al., 2023). Hence, in this study, we aimed to model the present and future potential habitat distribution of *N. pallida* within the dry forests of Peru, elucidating the relationship between the species and its habitat through response curves derived from key environmental variables. The primary research objectives included: (1) assessing the current and future potential distribution of *N. pallida* in Peru under different climate scenarios, (2) investigating the influence of environmental factors on the spatial distribution of *N. pallida* habitat, and (3) understanding how climate change will impact the future distribution and spatial patterns of *N. pallida*. Addressing these inquiries can not only provide theoretical support for strategic planning related to the introduction and cultivation of *N. pallida* in Peru but also establish a scientific foundation for its restoration and conservation efforts.

## 2 METHODS

### 2.1 Study area

**FIGURE 1:**
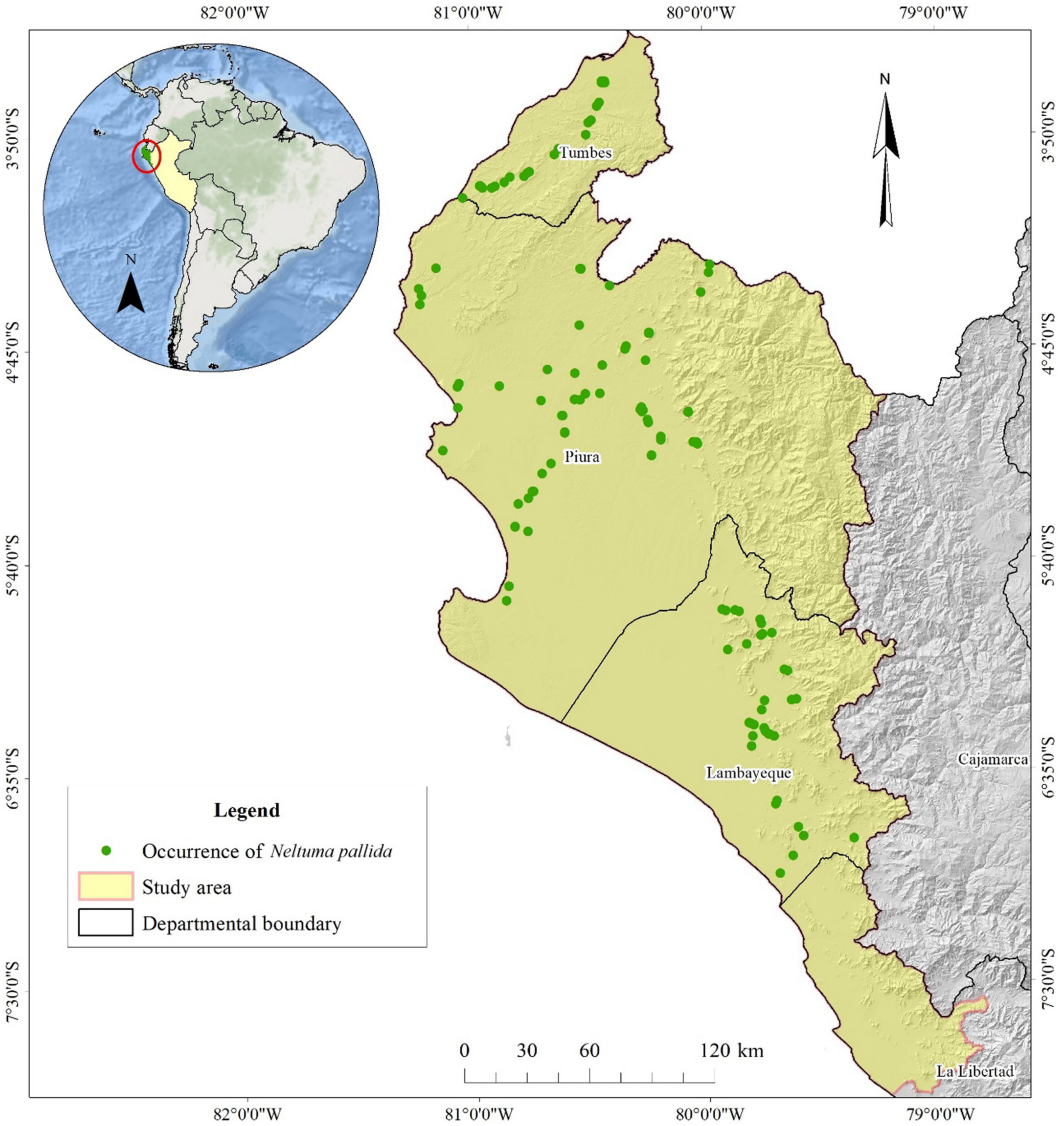
The study area for *Neltuma pallida*, along with the boundaries of the different geographical departments and the georeferenced records.

The study area consisted of the Equatorial Dry Forest, located in the geographical departments of La Libertad, Lambayeque, Piura, and Tumbes in northern Peru (Figure 1) with an altitudinal range that varies from 0 to 1500 masl (Salazar et al., 2021). The mean annual precipitation ranges from 100 to 500 mm with the months of highest rainfall from January to March, in turn, the mean annual temperature varies from 24 to 27°C and is directly correlated with the intensity of rainfall and the thermal inertia of the Pacific Ocean (Rollenbeck et al., 2015). The study area presents a flat to semi‐flat topography with soils originating from eolian or alluvial deposition (Salazar, Navarro‐Cerrillo, Cruz, & Villar, 2018). The study area comprises the Sechura desert, agricultural surfaces and forests where *Loxopterygium huasango* (Hualtaco), *Neltuma* spp. (Algarrobo), *Bursera graveolens* (Palo santo), *Eriotheca ruizii* (Pasayo), *Capparis scabrida* (Sapote), and *Cordia lutea* (Overo) species predominate in the arboreal stratum (Barboza et al., 2022; Caycho et al., 2023; SERFOR, 2020).

**FIGURE 2:**
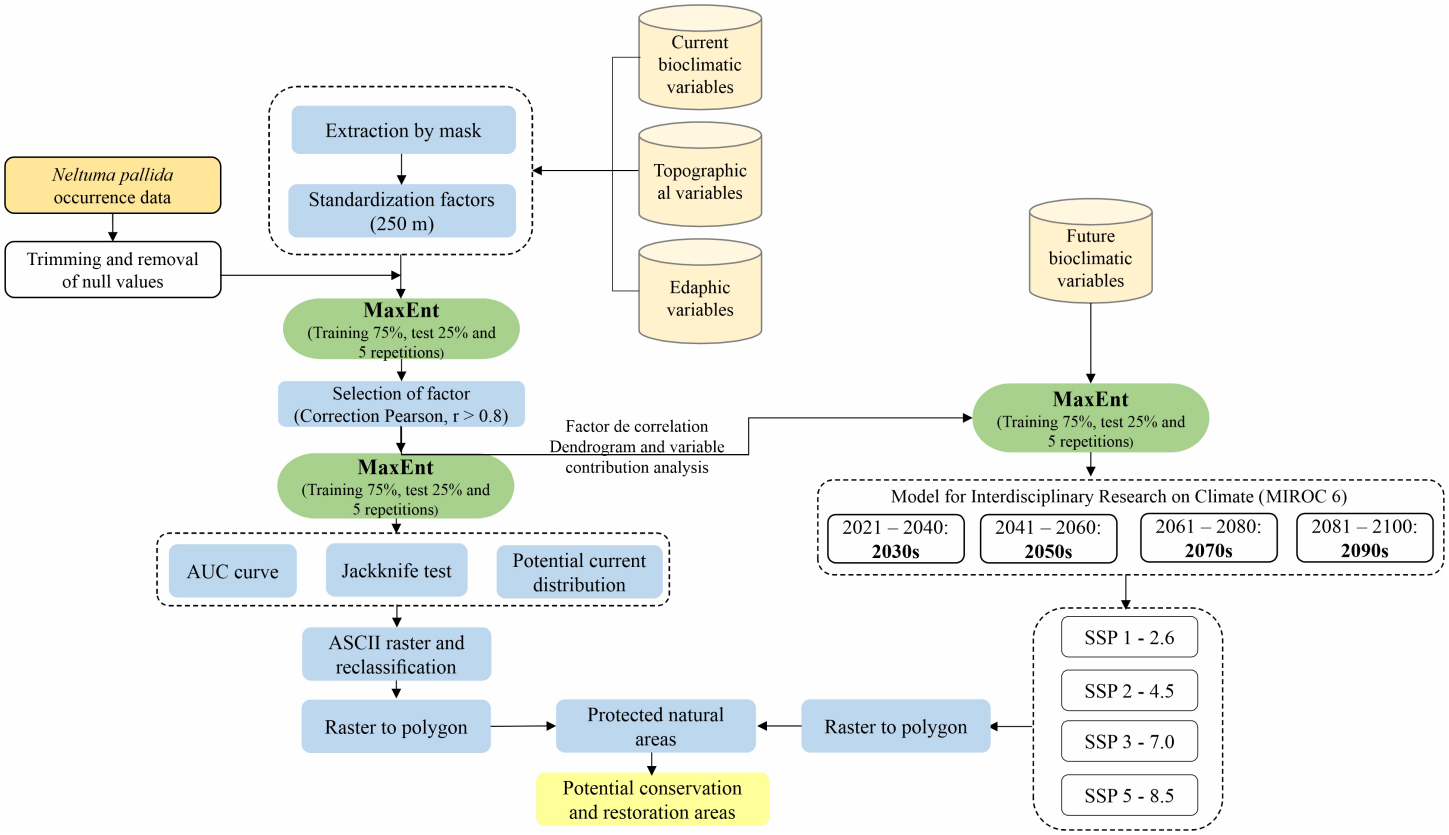
Flowchart outlining the methodology to evaluate the spatial modeling of the present and future distribution of *Neltuma pallida*.

Figure 2 describes the methodological flowchart to analyze the current and future spatial distribution of the *N. pallida*, based on the collection, cutting, and standardization of bioclimatic, topographic, and edaphic variables according to the study area. Likewise, the information on georeferenced presence data of *N. pallida* was collected. Finally, the MaxEnt algorithm was applied to model the current and future potential distribution for 2100 using the Model for Interdisciplinary Research on Climate (MIROC 6) and the different Shared Socioeconomic Pathways (SSP).

### 2.2 Source of data for the presence of *N. pallida*

The 132 data points on the presence of *N. pallida*, duly georeferenced, were retrieved (longitude, latitude, and altitude) in CSV format, from the fieldwork that was carried out from June 15 to June 22, 2002 and downloaded from the GBIF only for the study area (https://www.gbif.org/, accessed September 24, 2022). Those lacking exact geographic coordinates were corrected through visual interpretation in Google Earth Pro (Zhang et al., 2021). Likewise, spatial analysis was applied in QGIS v. 3.16 to decrease the impact of spatial autocorrelation, ensuring that each pixel contains a single point of presence (Liu et al., 2021; Yang et al., 2022).

### 2.3 Environmental variables

Environmental factors such as soil, climate, and topography play a key role in the development and distribution of flora (Yang et al., 2022). To address these considerations, a total of 36 variables were chosen (Table 1). Twenty‐three of these variables constituted bioclimatic data for the current period spanning 1970–2000 (Fick & Hijmans, 2017), while terrain‐related factors, specifically altitude, slope, flow direction, terrain roughness index, and topographical position index were obtained from digital elevation model (Hennig et al., 2007), downloaded from WorldClim 2.1 data set (https://www.worldclim.org/) and procesing in Google Earth Engine (GEE) (Gorelick et al., 2017). The remaining eigth variables, relating to soil properties, were obtained at 30 arcsecond (~1 km) resolution from the SoilGrids 0.5.3 database (http://soilgrids.org) using GEE.

**TABLE 1:** Variables used for current and future modeling of *Neltuma pallida* in MaxEnt.

Variables		Units	Symbols
1. Bioclimatic	Average annual temperature	°C	bio01
	Avearge diurnal range	°C	bio02
	Isothermality		bio03
	Temperature seasonality	°C	bio04
	Maximum temperature of warmest month	°C	bio05
	Minimum temperature of coldest month	°C	bio06
	Annual temperature range	°C	bio07
	Average temperature of wettest quarter	°C	bio08
	Average temperature of driest quarter	°C	bio09
	Average temperature of warmest quarter	°C	bio10
	Average temperature of coldest quarter	°C	bio11
	Annual precipitation	mm	bio12
	Precipitation of rainiest month	mm	bio13
	Rainfall of driest month	mm	bio14
	Precipitation seasonality	mm	bio15
	Rainfall of wettest quarter	mm	bio16
	Rainfall of driest quarter	mm	bio17
	Precipitation of warmest quarter	mm	bio18
	Precipitation of coldest quarter	mm	bio19
	Minimum temperature	°C	Tem_min
	Maximum temperature	°C	Tem_max
	Average temperature	°C	Tem_mean
	Precipitation	mm	Prec
2. Topographic	Elevation above sea level	masl	dem
	Land slope	°	slope
	Flow direction		flowd
	Terrain Roughness Index—TRI		TRI
	Topographical Position Index—TPI		TPI
Edaphic at 0.60 m	pH in H_2_O	pH × 10	pH
	Soil organic carbon content in fine soil fraction	g/kg	soc
	Bulk density of fine soil fraction	kg/dm^3^	bdod
	Total nitrogen (N)	g/kg	nitrogen
	Clay content	%	clay
	Sand content	%	sand
	Silt content	%	slime
	Carbon stock	kg/m^2^	ocs

The bioclimatic, topographic, and edaphic variables were processed at 250 m resolution. To determine the importance of each variable, the Jackknife method was applied (Meza et al., 2022). Likewise, Pearson's correlation was employed to overcome collinearity between the variables (Aidoo et al., 2022; Dormann et al., 2013; Leroy et al., 2016; Owens et al., 2013). The “virtual species” package in R Studio was applied to process them (Leroy et al., 2016), and the variables were grouped by clustering, with Pearson's correlations greater than 0.80 (Cotrina, Rojas, et al., 2021; Wei et al., 2018). The establishment of a high‐performance model was carried out with the selection of 10 variables (bio3, bio4, bio6, bio8, bio9, bio12, bio15, altitude, slope, flow direction).

### 2.4 Future scenarios of climate change

We use future bioclimatic data from the sixth version of the MIROC 6 (Tatebe et al., 2019). It provides information related to climate predictions from seasonal to decadal, future climate projections, being widely used at different scales (Coulibaly et al., 2023; Hirabayashi et al., 2022; Kunwar et al., 2023; SERFOR, 2020; Sharma et al., 2018). Climate data were obtained from the Coupled Model Intercomparison Project (CMIP) multimodel under the six least and most extreme shared socioeconomic pathways (SSP) (SSP1–2.6, SSP2–4.5, SSP3–7.0, SSP4–8.5), for projections to 2021–2040, 2041–2060, 2061–2080, 2081–2100 (denoted years 2030, 2050, 2070, and 2090) from Worclim (https://www.worldclim.org/data/cmip6/cmip6climate.html).

### 2.5 Model execution

The biogeographic distribution model for *N. pallida* was constructed using MaxEnt. This model assessed the likelihood of potential distribution for each species based on the presence data (locations) (OSINFOR, 2013, 2016). To validate this model, 75% of randomly selected presence data points were used for training, while the remaining 25% were set aside for validation, as outlined by Liu et al. (2021).

The resulting model was subjected to validation based on the area under the curve (AUC), which was calculated using the receiver‐operating characteristic (ROC) method and categorized into levels ranging from invalid (<0.6) to excellent (>0.9) (Peterson et al., 2008, 2011). To create a model of assessed species, the logistic output format was used, generating a plot with continuous values ranging from 0 to 1. This plot was subsequently reclassified into four habitat categories: (1) high potential (>0.6), (2) moderate (0.4–0.6), (3) low potential (0.2–0.4), and (4) no potential (0.5–0.5) (Meza et al., 2022; Zhang et al., 2021).

### 2.6 Suitable habitat classification

To improve the performance of the model, five replications of cross‐validation were done (Liu et al., 2021). Subsequently, according to the results of habitat levels for each scenario, the maps were reclassified into four habitats suitable categories: highly (0.5 ≤ *p* ≤ 1.0), moderately (0.3 ≤ *p* < .5), little (0.1 ≤ *p* < .3), and unsuitable (*p* < .1).

## 3 RESULTS

**FIGURE 3:**
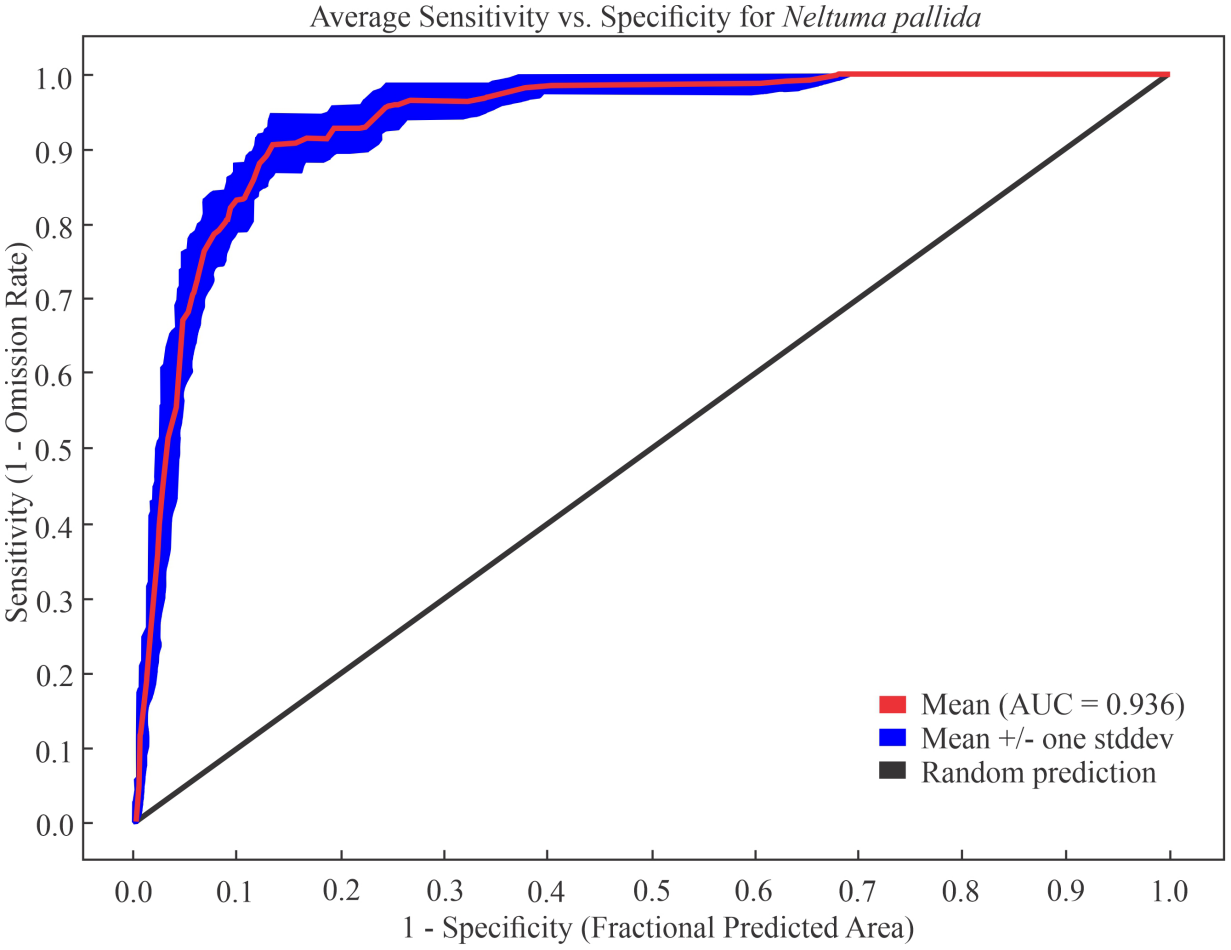
Reliability test of the distribution model based on area under the curve (AUC) and mean sensitivity versus specificity for *Neltuma pallida*.

Table 2 shows the results of the statistical method of the ROC curve that allowed to compare the average sensitivity with the specificity of *N. pallida* in current and future conditions. For the current distribution, average of the five repetitions reported an AUC of 0.94, and a standard deviation is 0.014 (Figure 3), while, for future scenarios, the AUC ranged between 0.93 and 0.95 (Table 2).

**TABLE 2:** Assessment of the species distribution model's performance (measured by AUC) in both the current environmental conditions and various climate change scenarios.

Representation	AUC			
Current	0.94			
MIROC6	SSP1–2.6	SSP2–4.5	SSP3–7.0	SSP5–8.5
2030s	0.94	0.93	0.94	0.93
2050s	0.95	0.93	0.93	0.94
2070s	0.93	0.95	0.93	0.93
2090s	0.94	0.93	0.94	0.93

Abbreviation: AUC, area under the curve.

### 3.1 Precision model and current distribution

**FIGURE 4:**
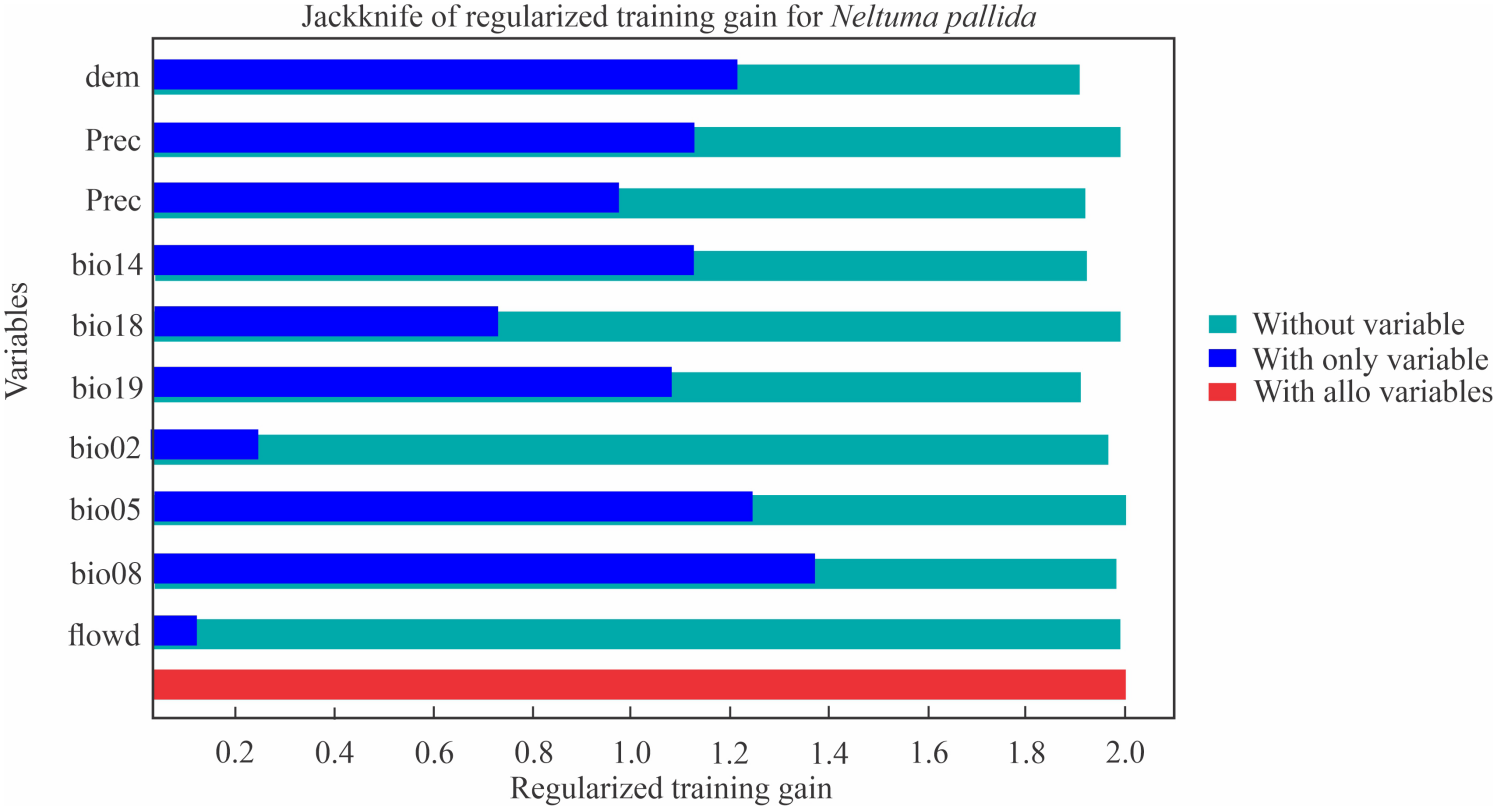
Relative contributions of the variables to the MaxEnt model to assess the potential habitat distribution of *Neltuma pallida*.

The Jackknife test results indicated that the primary factors influencing the potential habitat distribution of *N. pallida* were the mean temperature during the wettest quarter (bio08), the maximum temperature in the warmest month (bio05), altitude (dem), precipitation, and precipitation during the driest month (bio14), as illustrated in Figure 4.

**FIGURE 5:**
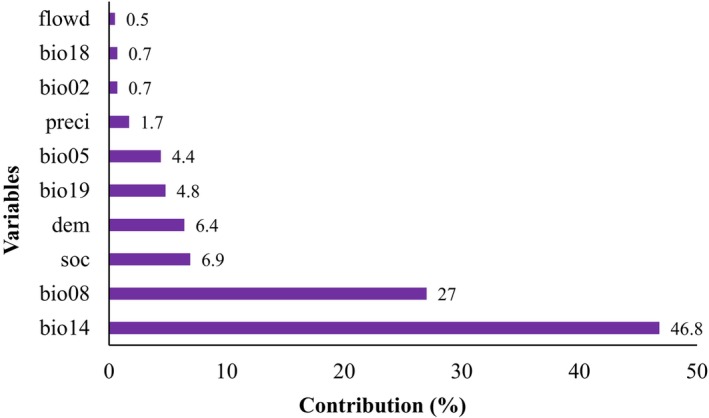
Analysis of variable contributions to the MaxEnt model to assess the potential habitat distribution of *Neltuma pallida*.

The modeling reported that the relative contributions of the two variables, the bio14 and the bio08, were the most influenced on the distribution of *N. pallida*, which explained 46.8% and 27% of the species habitat distribution, respectively. On the other hand, the variables of least contribution were the bio02, the bio18, and the flow direction with 0.7%, 0.7%, and 0.5%, respectively (Figure 5).

**FIGURE 6:**
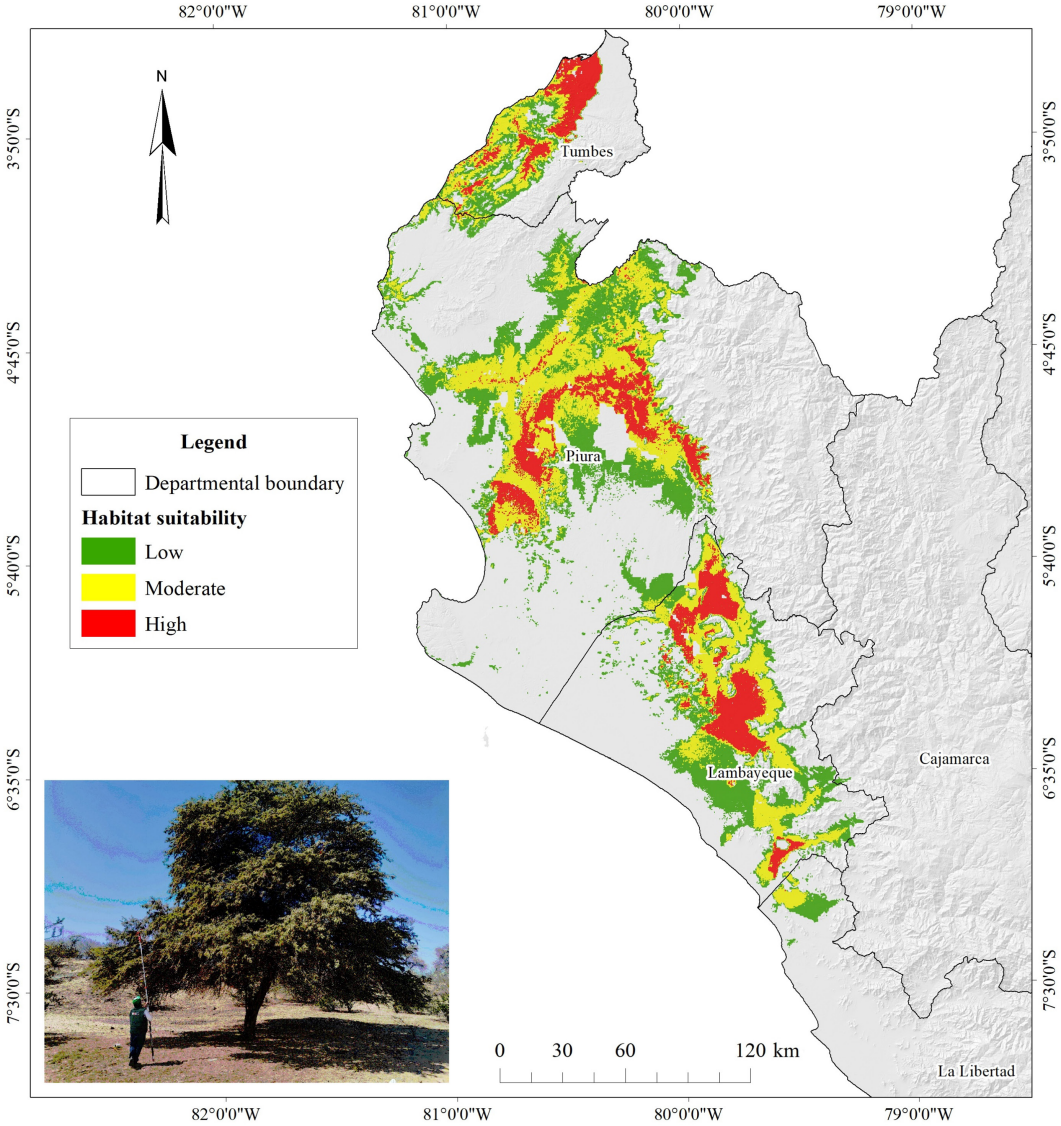
Current distribution of *Neltuma pallida*.

The current potential distribution of *N. pallida* was located throughout the study area from north to south, stretching the Tumbes, Piura, Lambayeque, and La Libertad departments (Figure 6). The “highly suitable” potential habitat represented 3.45% (68,304.32 km^2^), the “moderately suitable” hábitat 8.03% (6910.93 km^2^), the “low” habitat 9.12% (7842.13 km^2^), and the “not suitable” habitat 79.39% (68,304.32 km^2^).

### 3.2 Potential distribution of *N. pallida* under future climate scenarios

The unsuitable areas in the Equatorial Dry Forest of Peru for *N. pallida* are likely to be reduced in the coming years to become areas of “low,” “medium,” and “high” potential habitats. Table 3 shows that the “low” potential habitat in the future scenarios will increase by 12.78, 11.34, 10.16, and 11.65% by 2030 (SSP5‐8.5), 2050 (SSP5‐8.5), 2070 (SSP5‐8.5), and 2090 (SSP2‐4.5), respectively.

**TABLE 3:** Current distribution range of *Neltuma pallida* and the percentage of change in various scenarios in northern Peru.

Climatic scenarios	Períod	Unsuitable		Low		Moderate		High	
		km^2^	%	km^2^	%	km^2^	%	km^2^	%
Current	1970–2000	42,290.08	79.39	7842.13	9.12	6910.93	8.03	2975.70	3.46
2021–2040 (2030s)	SSP1–2.6	38,272.20	74.72 (−4.67)	9048.63	10.52 (1.40)	7658.31	8.90 (0.87)	5039.71	5.86 (2.40)
	SSP2–4.5	38,925.10	75.48 (−3.91)	8506.20	9.89 (0.77)	7942.07	9.23 (1.20)	4645.47	5.40 (1.94)
	SSP3–7.0	38,170.53	74.60 (−4.79)	9300.23	10.81 (1.69)	7826.36	9.10 (1.06)	4721.73	5.49 (2.03)
	SSP5–8.5	36,867.35	73.09 (−6.30)	10,995.19	12.78 (3.66)	7479.46	8.69 (0.66)	4676.84	5.44 (1.98)
2041–2060 (2050s)	SSP1–2.6	38,706.00	75.23 (−4.17)	9122.40	10.60 (1.49)	7397.95	8.60 (0.57)	4792.50	5.57 (2.11)
	SSP2–4.5	38,224.24	74.67 (−4.73)	9584.68	11.14 (2.03)	7640.12	8.88 (0.85)	4569.80	5.31 (1.85)
	SSP3–7.0	37,649.37	74.00 (−5.39)	9067.57	10.54 (1.42)	8645.90	10.05–(2.02)	4656.01	5.41 (1.95)
	SSP5–8.5	38,184.89	74.62 (−4.77)	9759.63	11.34 (2.23)	7610.87	8.85 (0.81)	4463.46	5.19 (1.73)
2061–2080 (2070s)	SSP1–2.6	39,442.83	76.08 (−3.31)	8085.31	9.40 (0.28)	8201.18	9.53 (1.50)	4289.53	4.99 (1.53)
	SSP2–4.5	39,552.81	76.21(−3.18)	8544.53	9.93 (0.82)	7732.26	8.99 (0.95)	4189.25	4.87 (1.41)
	SSP3–7.0	42,457.93	79.59 (0.20)	7793.18	9.06 (0.06)	6179.67	7.18 (0.85)	3588.08	4.17 (0.71)
	SSP5–8.5	39,274.54	75.89 (−3.51)	8743.56	10.16 (1.05)	7641.68	8.88 (0.85)	4359.07	5.07 (1.61)
2081–2100 (2090s)	SSP1–2.6	38,911.73	75.47 (−3.93)	9193.27	10.69 (1.57)	7107.63	8.26 (0.23)	4806.23	5.59 (2.13)
	SSP2–4.5	36,888.46	73.11 (−6.28)	10,026.54	11.65 (2.54)	8346.75	9.70 (1.67)	4757.10	5.53 (2.07)
	SSP3–7.0	37,321.61	73.62 (−5.78)	9425.35	10.96 (1.84)	7959.02	9.25 (1.22)	5312.87	6.18 (2.72)
	SSP5–8.5	39,487.01	76.13 (−3.26)	8229.17	9.57 (0.45)	7787.74	9.05 (1.02)	4514.93	5.25 (1.79)

The “moderate” potential habitat distribution reported by 2030s will increase with respect to current conditions by 9.10, 10.05, 9.53, and 9.70% according to (SSP3–7.0), 2050s (SSP3–7.0), 2070s (SSP1–2.6), and 2090s (SSP2–4.5), respectively. In turn, the spatial distribution of the “high” potential habitat showed similar conditions to the previous ones, with an area increase of 5.86, 5.57, 5.07, and 6.18% by 2030s (SSP1–2.6), 2050s (SSP1–2.6), 2070s (SSP5–8.5), and 2090s (SSP3–7.0), respectively.

**FIGURE 7:**
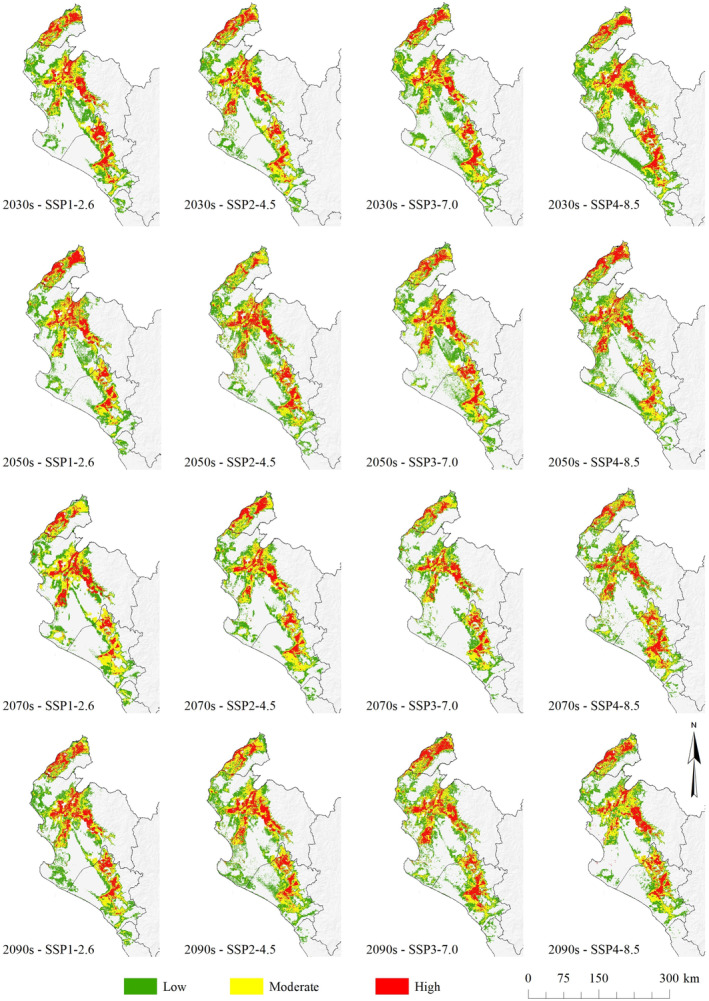
Future distribution of suitable areas for *Neltuma pallida* under various climate change scenarios.

The potential current distribution of *N. pallida* overlapped with the future distribution under climate change scenarios (Figure 7), an habitat increase was observed by 2100 at all potentiality levels. The results showed that habitat characteristics are relatively consistent across the four SSPs, in the different forecast years. The high future potential is in the central part of the geographical departaments of Tumbes, Piura, and Lambayeque, distributed from north to south.

**FIGURE 8:**
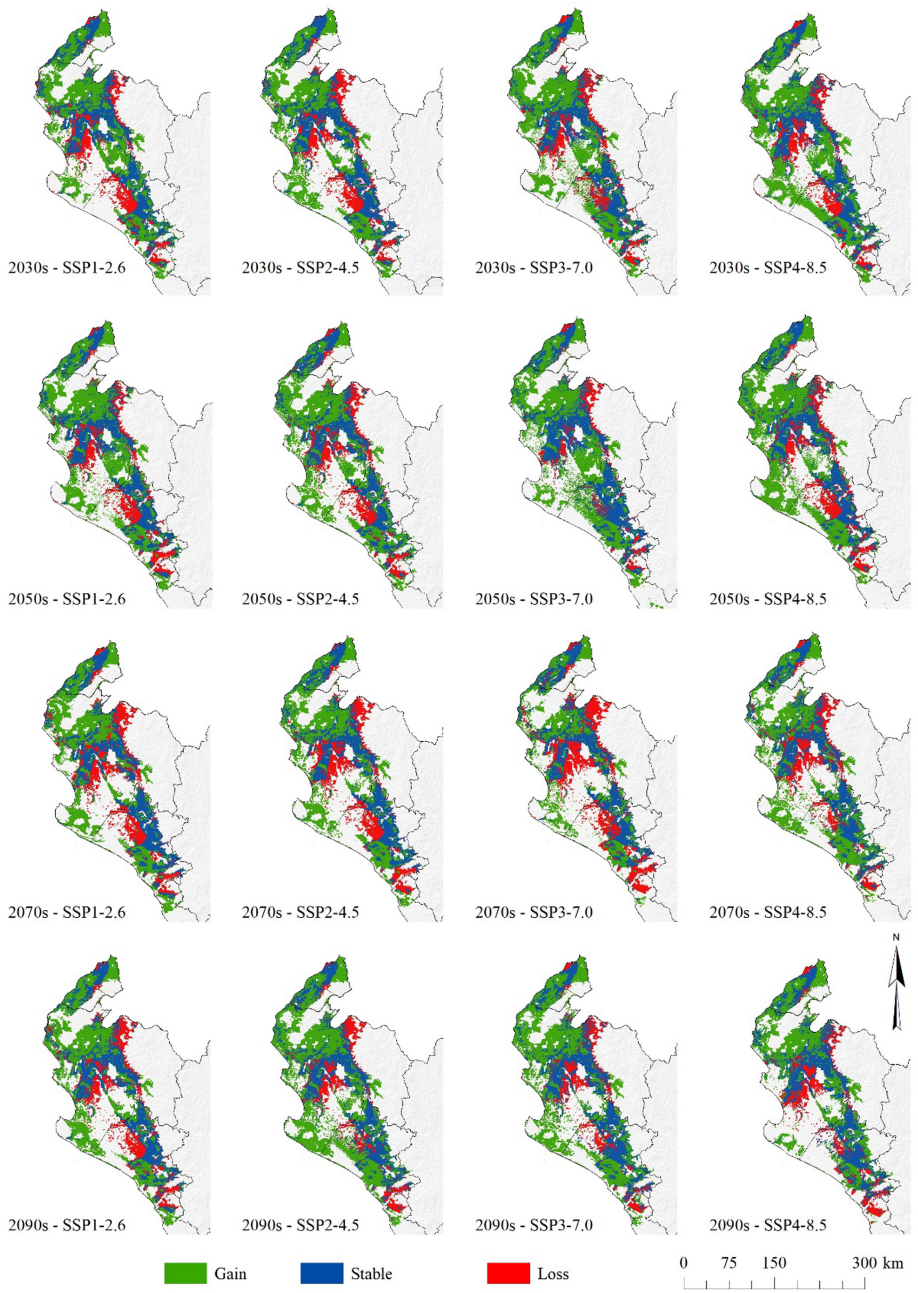
Distribution of potential habitat loss, gain, or permanence of *Neltuma pallida* habitat under climate change scenarios.

The potential distribution of the *N. pallida* current habitat overlapped with climate change scenarios to obtain characteristics of habitat loss, gain, or permanence spatially (Figure 8). The habitat areas of *N. pallida* showed a trend of area gain by 2100. The areas of surface gain were located mainly in the southwest, center‐west, and north of the study area. On the other hand, the loss zones were located mainly in the center and center‐south of the study area. In addition, the most stable zones under climate change scenarios are located in the central part of the dry forest.

## 4 DISCUSSION

The performance results of MaxEnt indicated high precision and reliability, as the AUC values ranged between 0.93 and 0.95. If the value exceeds this threshold, it represents exceptionally high predictive accuracy of the model (Swets, 1988). The current zones with “high,” “moderate,” and “low” potential are distributed in the central areas of the departments of Tumbes, Piura, and Lambayeque, as well as in the northern part of La Libertad, central to the study area. This study reports an increase in the surface areas from current to future conditions of *N. pallida*. The results are similar to those reported by Oliveira et al. (2018), who analyzed the dynamics of climatic niches of *Prosopis juliflora* (now *N. juliflora*) and *Prosopis pallida* (now *N. pallida*) in semi‐arid areas of Brazil. A similar trend is observed in Ethiopia, where the current and potential distribution of *N. juliflora* was mapped, reporting an increase in surface area (Wakie et al., 2014). The use of satellite images such as Landsat 8 and Sentinel‐2 has also contributed to mapping *N. juliflora* and demonstraated its long‐term surface increase under climate change scenarios (Ahmed et al., 2021; Rembold et al., 2015). However, other species of dry forests could be affected in future scenarios, such as *Anadenanthera colubrina*, *Aspidosperma pyrifolium*, and *Myracrodruon urundeuva* (Rodrigues et al., 2015), as well as cacti (Cavalcante & Sampaio, 2022), *Calycophyllum multiflorum* (Alabar et al., 2022), *Albizia multiflora*, *Ceiba trichistandra*, and *Cochlospermum vitifolium* (Manchego et al., 2017).

The increase in the habitat distribution of *N. pallida* in the study area could be related to its adaptability to extremely dry and wet conditions. This climatic event creates two alternative states that promote survival and growth strategies, respectively (Holmgren et al., 2001; Salazar, Navarro‐Cerrillo, Cruz, & Villar, 2018). Unlike other species of dry forests, these changes have allowed the species to develop physiological and morphological adaptations to survive in years of drought, grow after floods, and in saline soils. This adaptability appears to be strongly associated with the occurrence of El Niño on the northern coast of Peru (Palacios et al., 2012; Salazar et al., 2021). However, *N. pallida* is considered an invasive species in some areas such as Australia, Africa, Ethiopia, Brazil, and Pacific Islands (Ahmed et al., 2021; Gallaher & Merlin, 2010; Rembold et al., 2015; Sintayehu et al., 2020). The decline in *N. pallida* conditions may be related to pest and disease attacks, forest fires, and indiscriminate logging.

**FIGURE 9:**
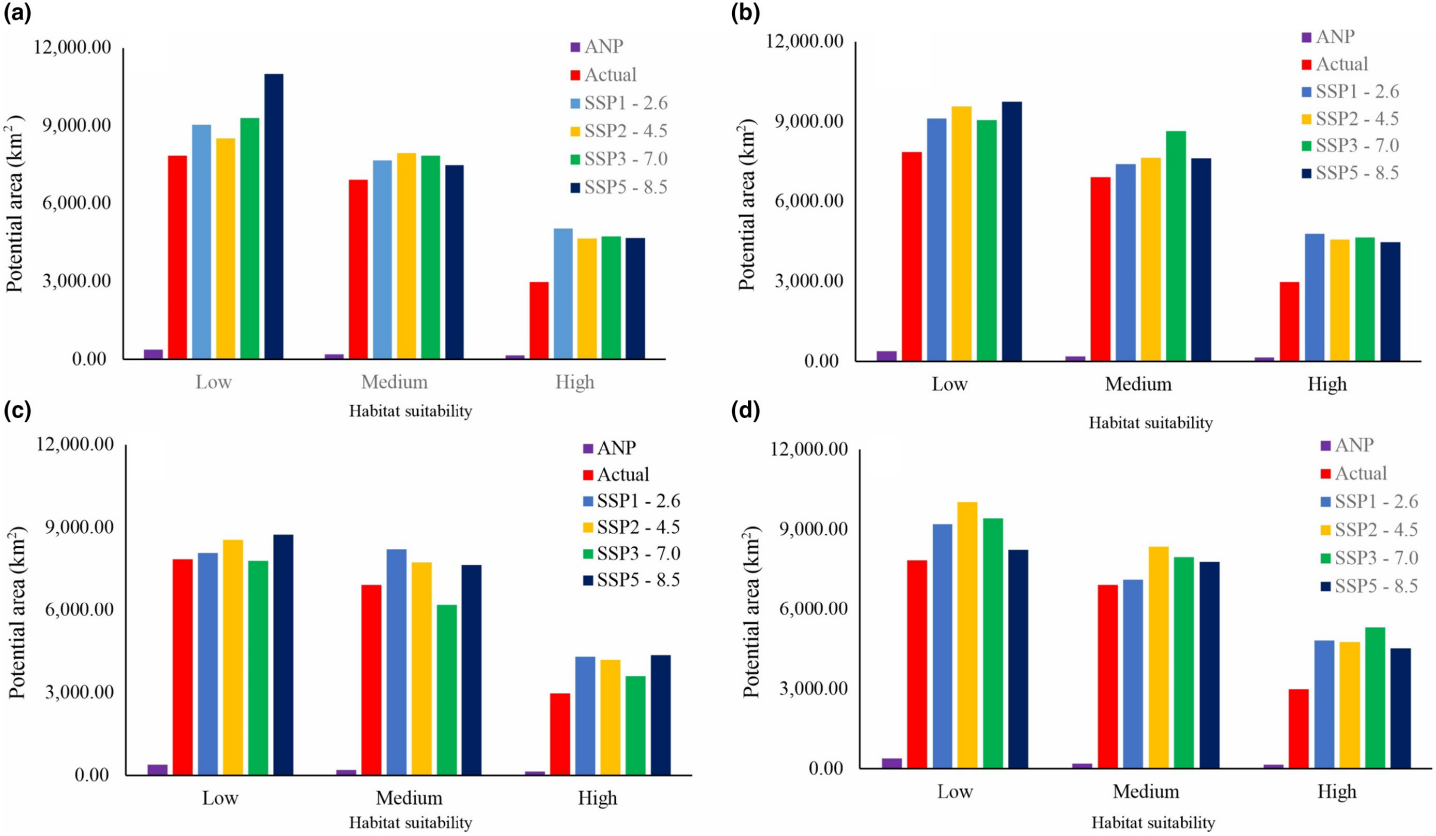
Distribution of the conserved area of *Neltuma pallida* under different climate change scenarios, according to habitat levels (a) 2030s, (b) 2050s, (c) 2070s, and (d) 2090s.

Within the arid forests of Peru, there is a variety of species, highlighting the need to adopt effective strategies aimed at their monitoring and preservation, as emphasized by Cotrina, Bandopadhyay, et al. (2021). *N. pallida* is one of the main species in this ecosystem; however, it is threatened by anthropogenic activities such as deforestation, land use change, and logging (Nieuwstadt & Sheil, 2005; Qarallah et al., 2021). Efforts are currently underway to conserve the biodiversity of the ecosystem and *N. pallida* individuals through the creation of protected areas (725.69 km^2^, Figure 9) (SERNAP, 2023). The conservation of these species is vital because they provide ecosystem services that contribute to people's economy. Therefore, it is essential to implement measures that help mitigate the species' population decline and ensure its sustainable use (INIA, 2020).

This research determined the current and future habitat distribution areas of *N. pallida*. Based on this, potential species conservation areas were identified, and conservation plans were established (Li et al., 2020). Additionally, future studies could utilize other climatic models, such as the fusion of methodologies like Analytic Hierarchy Process (AHP), GEE, and GARP (Cotrina, Bandopadhyay, et al., 2021; Padalia et al., 2014; Rojas‐Briceño et al., 2022; Zhang et al., 2021). In this research, we integrated tools like QGIS, Rstudio, GEE, and MaxEnt to identify potential distribution zones of *N. pallida* under current and future conditions, which yielded favorable results. Although MaxEnt is one of the most used models, it has certain disadvantages in considering climatic impact, topographical, and environmental factors (nonbiological factors), in areas heavily impacted by human activities (Wang et al., 2020). Therefore, this model only represents potential habitat distribution rather than real distribution (Liu et al., 2021). A detailed and predictive analysis of the future potential habitat distribution of species could be crucial for developing conservation programs and ensuring the species' survival probability against the effects of climate change (Khanal et al., 2022).

## 5 CONCLUSIONS

The area of *N. pallida* in northern Peru covers approximately 17,728.76 km^2^ distributed among the geographical departments of Tumbes, Piura, Lambayeque, and La Libertad. Also, the area conserved through protected natural areas represents 2.23% of the distribution of this species. The critical factors influencing the distribution of *N. pallida* are related to the average temperature during the wettest quarter, the maximum temperature in the warmest month, altitude, precipitation, and precipitation levels during the driest month. In turn, under climate change scenarios to 2100, the potential “high” distribution of the species showed an increase ranging from 3.56% to 5.25%, especially in the SSP5–8.5 scenario. These current and future distribution maps may help identify new areas for the design of conservation and population recovery strategies with a focus on ecological restoration as these areas are expected to have suitable conditions for the development of the species.

## AUTHOR CONTRIBUTIONS

**Elgar Barboza:** Conceptualization (lead); data curation (lead); formal analysis (lead); investigation (equal); methodology (equal); software (equal); visualization (equal); writing – original draft (lead); writing – review and editing (lead). **Nino Bravo:** Conceptualization (equal); data curation (equal); formal analysis (equal); investigation (equal); methodology (equal); writing – review and editing (equal). **Alexander Cotrina‐Sanchez:** Data curation (equal); formal analysis (equal); investigation (equal); methodology (equal); writing – review and editing (equal). **Wilian Salazar:** Formal analysis (equal); investigation (equal); methodology (equal); supervision (equal); writing – review and editing (equal). **David Gálvez‐Paucar:** Conceptualization (equal); investigation (equal); visualization (equal); writing – review and editing (equal). **Jhony Gonzales:** Conceptualization (equal); investigation (equal); supervision (equal); visualization (equal); writing – review and editing (equal). **David Saravia:** Investigation (equal); methodology (equal); visualization (equal); writing – review and editing (equal). **Lamberto Valqui‐Valqui:** Investigation (equal); methodology (equal); visualization (equal); writing – review and editing (equal). **Gloria P. Cárdenas:** Investigation (equal); methodology (equal); writing – review and editing (equal). **Jimmy Ocaña:** Investigation (equal); methodology (equal); writing – review and editing (equal). **Juancarlos Cruz‐Luis:** Funding acquisition (equal); project administration (equal); supervision (equal); visualization (equal); writing – review and editing (equal). **Carlos I. Arbizu:** Conceptualization (equal); funding acquisition (equal); project administration (equal); supervision (equal); visualization (equal); writing – review and editing (equal).

## FUNDING INFORMATION

This research was funded by the following three research projects of the Peruvian Government: (i) “Creación del servicio de agricultura de precisión en los Departamentos de Lambayeque, Huancavelica, Ucayali y San Martín 4 Departamentos,” (ii) “Mejoramiento de los servicios de investigación y transferencia tecnológica en el manejo y recuperación de suelos agrícolas degradados y aguas para riego en la pequeña y mediana agricultura en los departamentos de Lima, Áncash, San Martín, Cajamarca, Lambayeque, Junín, Ayacucho, Arequipa, Puno y Ucayali,” and (iii) Creación del Servicio de laboratorio de Biología Molecular para la Investigación en la Universidad Nacional de Frontera–Distrito de Sullana, with grant numbers CUI 2449640, CUI 2487112, and CUI 2437731, respectively.

## CONFLICT OF INTEREST STATEMENT

The authors declare no conflict of interest or competing interests.

## Data Availability

Data for the current manuscript is available at https://datadryad.org/stash/share/0vbaKZ1FTUF‐alH36dpQQ7gVQrDw0g_mqrdMdO8s9bc.
